# Operating at the very low end of the crassulacean acid metabolism spectrum: *Sesuvium portulacastrum* (Aizoaceae)

**DOI:** 10.1093/jxb/ery431

**Published:** 2018-12-07

**Authors:** Klaus Winter, Milton Garcia, Aurelio Virgo, Joseph A M Holtum

**Affiliations:** 1 Smithsonian Tropical Research Institute, Balboa, Ancón, Republic of Panama; 2 Centre for Tropical Biodiversity and Climate Change, College of Science and Engineering, James Cook University, Townsville, Queensland, Australia; 3 University of Essex, UK

**Keywords:** Crassulacean acid metabolism, CO_2_ assimilation, C_4_ photosynthesis, facultative CAM, salt tolerance, *Sesuvium portulacastrum*, succulence

## Abstract

Demonstration of crassulacean acid metabolism (CAM) in species with low usage of this system relative to C_3_-photosynthetic CO_2_ assimilation can be challenging experimentally but provides crucial information on the early steps of CAM evolution. Here, weakly expressed CAM was detected in the well-known pantropical coastal, leaf-succulent herb *Sesuvium portulacastrum*, demonstrating that CAM is present in the Sesuvioideae, the only sub-family of the Aizoaceae in which it had not yet been shown conclusively. In outdoor plots in Panama, leaves and stems of *S. portulacastrum* consistently exhibited a small degree of nocturnal acidification which, in leaves, increased during the dry season. In potted plants, nocturnal acidification was mainly facultative, as levels of acidification increased in a reversible manner following the imposition of short-term water-stress. In drought-stressed plants, nocturnal net CO_2_ exchange approached the CO_2_-compensation point, consistent with low rates of CO_2_ dark fixation sufficient to eliminate respiratory carbon loss. Detection of low-level CAM in *S. portulacastrum* adds to the growing number of species that cannot be considered C_3_ plants *sensu stricto*, although they obtain CO_2_ principally via the C_3_ pathway. Knowledge about the presence/absence of low-level CAM is critical when assessing trajectories of CAM evolution in lineages. The genus *Sesuvium* is of particular interest because it also contains C_4_ species.

## Teaser

Leaves and stems of the pantropical coastal herb *Sesuvium portulacastrum* exhibit low-level CAM that is enhanced by drought-stress, emphasizing the need for careful characterization of photosynthetic physiology when selecting species to study the evolution of photosynthetic pathways.

## Introduction

The Aizoaceae, a family of about 1600 species in the order Caryophyllales (Hartmann, 2017), contains more species with succulent leaves than any other eudicot family (Hernández-Ledesma *et al.*, 2015). In three of the four sub-families of the Aizoaceae, the Aizooideae, Mesembryanthemoideae, and the Ruschioideae, succulence is associated with multiple lineages of crassulacean acid metabolism (CAM), a water-use efficient pathway of photosynthesis (von Willert *et al.*, 1977, 1992; Smith and Winter, 1996; Arakaki *et al.*, 2011).

The Sesuvioideae, the earliest diverging sub-family (Klak *et al.*, 2003; Bohley *et al.*, 2015), is unique in the Aizoaceae in that it is the only sub-family within which CAM has not yet been demonstrated unequivocally (Bohley *et al.*, 2015) and it is the only sub-family that contains species with C_4_ photosynthesis, which is present in the genera *Sesuvium* (Bittrich, 1990; Bohley *et al.*, 2015), *Trianthema*, and *Zaleya* (Carolin *et al.*, 1978). Within *Sesuvium*, two clades have been recognized, one composed of species with C_4_-type anatomy, C_4_ biochemistry, and carbon isotope values of between –13.3 and –11.2‰ [*S. congense*, *S. crithmoides*, *S. humifusum* (formerly *Cypselea humifusum*), *S. mesembryanthemoides*, and *S. sesuvoides*) (Bohley *et al.*, 2015, 2017), and one with isotope values of between −27.7 and −25.3‰ that are characteristic of plants with C_3_ photosynthesis (*S. ayresii*, *S. dystylum*, *S. maritimum*, *S. microphyllum*, *S. portulacastrum*, *S. sessile*, and *S. verrucosum*) (Bohley *et al.*, 2015). For one species, *S. edmonstonei*, native to the Galapagos, a reported isotope value of −21.5‰ is intermediate between those characteristic of C_3_ plants and those of plants with C_4_ photosynthesis or full CAM (Winter and Holtum, 2002; Bohley *et al.*, 2015; Alonso-Cantabrana and von Caemmerer, 2016), although for plants of a northern Venezuelan population the δ^13^C value is –24‰ (Medina *et al.*, 2008).

Despite a lack of evidence for CAM-type CO_2_ dark fixation in the Sesuvioideae, the ~50 species in this small sub-family [*Sesuvium*, 14 spp. (Bohley *et al.*, 2017); *Trianthema*, ~20 spp. (Hartmann, 2017); *Tribulocarpus*, two spp. (Thulin *et al.*, 2012); *Zaleya*, six spp. (Gilbert *et al.*, 2000)] are mostly succulent-leaved and drought-tolerant, traits common in CAM plants (Ogburn and Edwards, 2010). Bearing in mind recent reports of low-level CAM in other small succulent-leaved herbs within the Caryophyllales that exhibit C_3_- or C_4_-type isotopic signatures [e.g. *Calandrinia* (Montiaceae), *Portulaca* (Portulacaceae), and in the succulent-leaved vine *Anredera baselloides* (Basellaceae)] (Holtum *et al.*, 2017*a*, 2017*b*, 2018; Winter and Holtum, 2017), we decided to test whether the ostensibly C_3_ or C_4_ isotope values reported for *Sesuvium* might mask the presence of low-level CAM. We were further encouraged to do so by a single observation of small nocturnal acidification in field-growing *S. maritimum* (Walter) Britton, Sterns & Poggenb. that exhibited a C_3_-type isotope value of –26‰ (Martin *et al.*, 1982).

A plant exhibits the CAM photosynthetic pathway when, in chloroplast-containing cells, CO_2_ is incorporated at night into malic acid that is stored in vacuoles (Osmond, 1978). In the subsequent light-period, the CO_2_ is retrieved from malic acid and is used for growth. The manifestation of CAM is therefore defined as an ability of a plant to fix CO_2_ in the dark and to accumulate acid at night. Nocturnal acidification is typically determined by measuring the differences in titratable acidity in extracts of tissue harvested at the beginning and the end of the night.

In the majority of plants with CAM, it is expressed alongside C_3_ photosynthesis or, in a few species, C_4_ photosynthesis (Koch and Kennedy, 1980; Winter and Holtum, 2017). The contribution of CAM to net daily carbon gain varies considerably between species. Nocturnal acidification reported for plants with CAM ranges over two orders of magnitude from 3–4 μmol H^+^ g^−1^ fresh mass (FM; close to the lower limit of detectability) to over 400 μmol H^+^ g^−1^ FM. At one extreme of the continuum, CAM may be the principal carbon-contributing pathway in a plant (Winter and Holtum, 2014), whereas at the other extreme the contribution of CAM to carbon gain may be extremely small [e.g. *Platycerium veitchii* (Holtum and Winter, 1999); *Jatropha curcas* (Winter and Holtum, 2015); *Yucca gloriosa* (Heyduk *et al.*, 2016)]. In the latter group, the machinery for CAM is nonetheless present and the pathway is operational, although nocturnal CO_2_ fixation may be masked by dark respiratory CO_2_ loss.

The expression of CAM may be constitutive, i.e. non-optional in that expression is part of the pre-set processes of development and growth, or it may be facultative, i.e. optional, in that it is not always present (Winter and Holtum, 2014; Winter *et al.*, 2015). Facultative CAM involves an induction or up-regulation of CAM in response to a stress (typically water stress) that is fully or largely lost when the stress is removed. Facultative and constitutive CAM are not necessarily mutually exclusive in a given photosynthetic organ. In a plant in which CAM is constitutively expressed, transient stress may transiently up-regulate CAM (Winter *et al.*, 2008). Because the contribution of dark CO_2_ fixation to lifetime carbon gain can be small in some facultative CAM plants and in species that exhibit low levels of constitutive CAM, such plants may exhibit isotope values similar to C_3_ or C_4_ plants and the detection of CAM in these species is best measured by quantifying both leaf acidification and nocturnal CO_2_ exchange during a wet-dry-wet sequence.

To further investigate the possibility of CAM in the Sesuvioideae we chose as a test species *S. portulacastrum*. Typically regarded as a C_3_ species even when growing under conditions of stress (Lüttge *et al.*, 1989; Voznesenskaya *et al.*, 2010; Bohley *et al.*, 2015), a single value of overnight H^+^ increase of field-growing *S. portulacastrum* has been reported (Ting, 1989). The species is a pan-tropical succulent-leaved coastal herb with an extensive distribution that spans coastlines of Africa, North and South America, tropical and temperate Asia, Australia, and the Pacific islands between 35°N and 42°S (Gonçalves, 1978; Lonard and Judd, 1997; Bohley *et al.*, 2017; Hartmann, 2017). In some regions, the salt-tolerating halophyte is cultivated for food and fodder, and used for dune stabilization and phytoremediation (Lokhande *et al.*, 2013*a*, 2013*b*).

Here, we provide results from a detailed study of gas exchange and nocturnal acidification in plants from a Panamanian population of *S. portulacastrum* either cultivated in pots or growing in outdoor experimental plots throughout tropical wet-season/dry-season transitions. Conclusive evidence is provided for the presence of low-level CAM in both the stems and leaves of *S. portulacastrum*.

## Materials and methods

### Plant material

Cuttings were obtained from five plants of *Sesuvium portulacastrum* (L.) L. collected at Sarigua National Park, Azuero Peninsula, Republic of Panama (8.013348N, 80.485658W) and further propagated at the Smithsonian Tropical Research Institute. A voucher specimen was deposited at the herbarium of the National University of Panama (PMA) (J. Aranda, A. Virgo, M. García, K. Winter 4324; 25 October 2018).

### Outdoor experiments

**Fig. 1.**
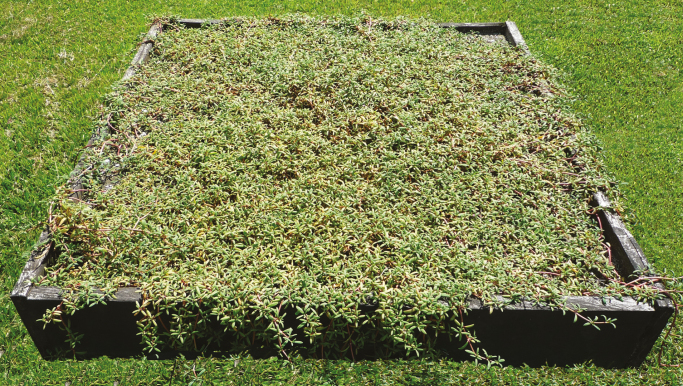
*Sesuvium portulacastrum* growing outside in a 1.5×1.5×0.3-m raised garden bed at the Smithsonian Tropical Research Institute, Republic of Panama in January 2018.

Two experiments were performed. In the first, at the beginning of September, 2016 (middle of the wet season), 10 cuttings were planted in forest soil in a 1.5×1.5×0.3 m raised garden bed surrounded by 3.5-cm thick wood panels at the Smithsonian Tropical Research Institute, Santa Cruz Experimental Research Facility, Gamboa, Republic of Panama (9.120085N, 79.701894W). Plants were exposed to full natural solar radiation and received natural rainfall. Plants were grown for 186 d. In the second experiment, which began on 1 June, 2017 (early wet season), plants derived from 25 cuttings were grown for 333 d in a mixture of 50:25:25% (v/v/v) of forest soil: potting mix (Miracle-Gro Lawn Products, Marysville, OH): sea-sand (Noveys, Panama) in a raised garden bed as described above (Fig. 1). From July to December 2017, in addition to natural rainfall, the plants received at monthly intervals 10 l of 10% seawater prepared from 3.5 g l^−1^ Ocean Salt (Instant Ocean, Blacksburg, VA (e.g. Reef *et al.*, 2016). Leaves and stems were sampled at dusk and dawn at weekly or 2-weekly intervals, unless specified otherwise. Samples were weighed for fresh mass determination and stored in liquid nitrogen to be processed further for measurements of titratable acidity, as described below.

### Pot experiments

Throughout 2016 and 2017, several experiments were conducted to study titratable acidity changes and gas-exchange responses during wet-dry-wet cycles. Plants were grown in terracotta pots (ranging from 0.5–3.5 l) containing potting mix (Miracle-Gro). Plants were maintained at the Tupper Centre of the Smithsonian Tropical Research Institute, Panama City (8.962568N, 79.543911W) under either full solar radiation or underneath rain shelters at 70% natural light. Growth conditions are specified in the relevant figure legends. As in the outdoor experiments, for acidity measurements mature leaves and stems excised from plants at dusk and dawn were weighed for determination of fresh mass and frozen in liquid nitrogen. In some experiments, prior to freezing the leaf area was determined using a LI-3100 leaf area meter (Li-Cor, Lincoln, NE).

### Titratable acidity and dry mass

Frozen tissue was either extracted sequentially in boiling 50% ethanol and in water (Winter and Holtum, 2017), or was freeze-dried for 72 h (Labonco, Freezeone 4.5, Kansas City, MO) and reweighed for dry mass determination before extraction. All extracts were titrated with 5 mM KOH to pH 6.5.

### Net CO_2_ exchange

To determine daily CO_2_ exchange patterns during wet-dry-wet cycles whole plants, attached branches, or individual attached leaves were studied. In one of the experiments, the shoot of a small plant was enclosed inside a Perspex cuvette (internal dimensions 11×11×10 cm) that rested on the 0.5-l terracotta pot in which the plant grew in potting mix (Miracle-Gro). The roots and the pot remained outside the cuvette. In another experiment, an individual leaf attached to a plant growing in a 1-l terracotta pot containing potting mix was enclosed in a 4.5-cm diameter PMK-10 leaf cuvette (porometer head; Walz, Effeltrich, FRG). After initial daily irrigation with water or a solution equivalent to 10% seawater, a drought treatment was imposed by withholding irrigation until net CO_2_ uptake during the light period approached zero, after which plants were rewatered daily.

The gas-exchange cuvettes were located inside a controlled environment chamber (GC8, EGC, Chagrin Falls, OH) operating under 12/12 h light/dark, 28/22 °C cycles. Illumination was supplied by a LED grow light (GroPro300, Model LL4L-GP300, www.LL4L.com). Photon flux densities are specified in the relevant figure legends. The cuvette was supplied with air containing 400 ppm CO_2_ at a flow rate of 1.26 l min^−1^. Net CO_2_ exchange was measured in a flow-through gas-exchange system consisting of Walz components and a LI-6252 CO_2_ Analyzer (Li-Cor) (Holtum and Winter, 2003).

## Results

### Outdoor experiments in garden beds

**Fig. 2.**
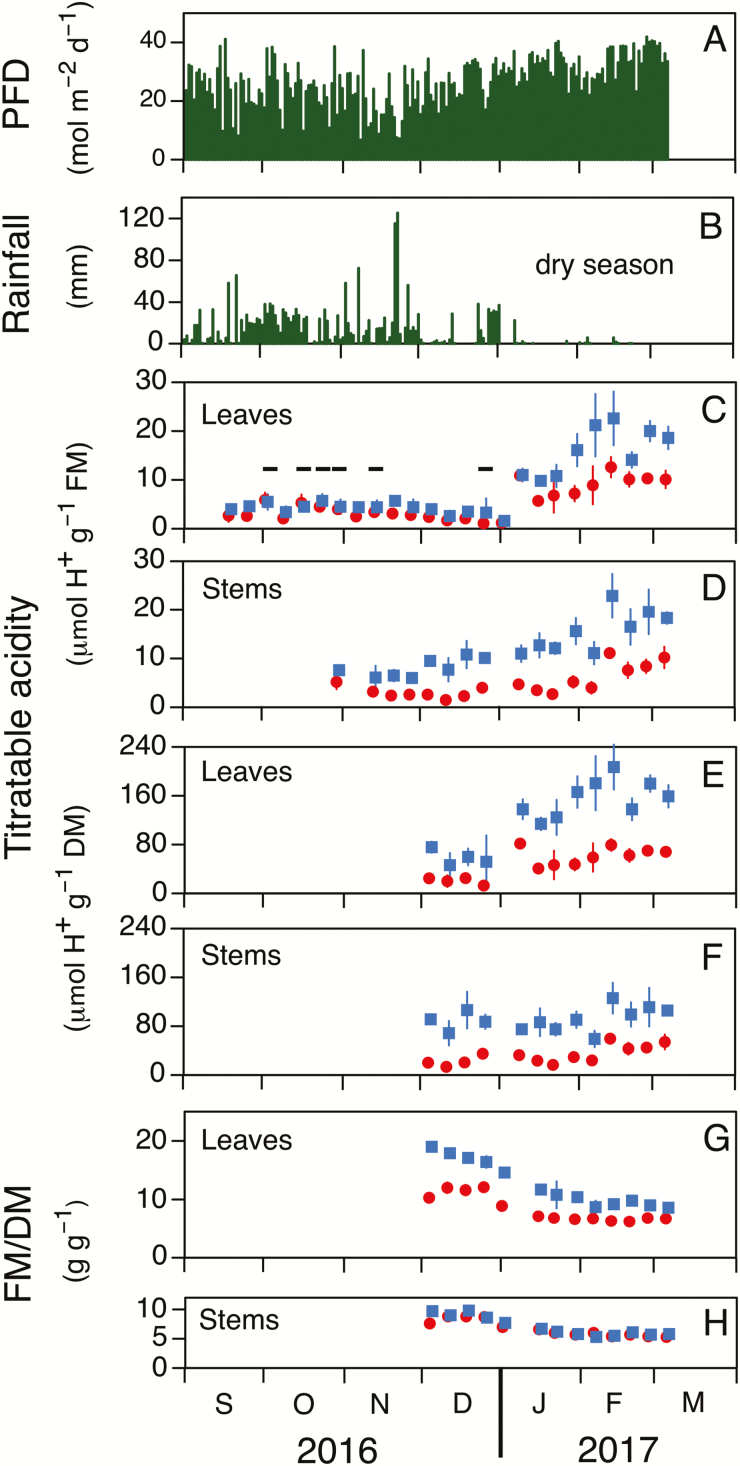
Seasonal changes in (A) photon flux density (PFD), (B) rainfall, (C–F) variation of titratable acidity at dusk (circles) and dawn (squares), and (G, H) variation in fresh:dry mass ratio (FM/DM) at dusk and dawn in leaves and stems of *Sesuvium portulacastrum*. Plants were grown in non-saline soil during the latter half of the 2016 wet season (September–December) and as they transitioned into the 2017 dry season. Acidity levels are expressed per unit fresh mass for leaves (C) and stems (D) and per unit dry mass for leaves (E) and stems (F). Acidity and FM/DM values are means (±SD) (*n*=5; for leaves, each sample comprised eight leaves). In (C–F), horizontal bars indicate non-significant differences between dusk and dawn values (one-tailed *t*-test, *P*≤0.05).

*Sesuvium portulacastrum* experienced two distinct climatic phases when grown outside for 186 d in forest soil (Fig. 2). During the first 125 d (i.e. from September 2016 to early January 2017) it rained on many days, whereas for the final 61 d rainfall was essentially absent (Fig. 2B). As rainfall ceased, the average daily irradiance increased (Fig. 2A). By the end of March, all plants had died. During the rainy period, leaves contained low levels of titratable acidity, when expressed on a fresh mass basis, but the values were significantly greater at dawn than at dusk for 9 of 15 weekly measurements (Fig. 2C). Stem acidity measurements began in October. In all wet-season samples, stem acidity increased significantly overnight, with the dawn-to-dusk difference being greater in stems than in leaves (Fig. 2C, D).

Soon after initiating the experiment, we noted that leaves slightly wilted during the daytime and recovered overnight. This observation prompted us to determine fresh:dry mass ratios from December onwards, and to express leaf and stem acidities also on a dry mass basis (Fig. 2E, F).

Nocturnal acidification in leaves, on both a fresh mass (FM) and a dry mass (DM) basis, was greater during the dry season than in the rainy season (Fig. 2C, E). ΔH^+^ was 1.3±0.9 μmol g^−1^ FM (mean±SD, *n*=15 d) during the wet season and 7.7±3.2 μmol g^−1^ FM (*n*=8 d) during the dry season (*P*≤0.001). Corresponding values on a dry mass basis were 38±10 μmol g^−1^ DM (*n*=4 d; wet season) and 95±26 μmol g^−1^ DM (*n*=9 d; dry season) (*P*≤0.001). In stems, acidification was also higher during the dry season, but only when values were expressed on a fresh mass basis (5.1±2.2 μmol g^−1^ FM, *n*=8 d, wet season versus 9.1±1.8 μmol g^−1^ FM, *n*=9 d, dry season; *P*≤0.001) and not when expressed on a dry mass basis (Fig. 2D, F). As the soil dried out during the progression between seasons, the FM to DM ratios of both leaves and stems decreased (Fig. 2G, H). Diurnal reductions in leaf FM:DM were large during the wet season and decreased during the dry season (Fig. 2G). Diurnal variations in FM:DM were largely absent in stems.

Since *S. portulacastrum* typically grows in coastal saline environments, a second outdoor experiment was conducted during the following 2017/2018 season in which plants were irrigated monthly with 10% seawater during the wet period. The input of extra moisture was small, equivalent to 4.4 mm precipitation per month. The salt was supplied to fulfil a hypothetical requirement for optimal growth, not to stress the plants *per se*.

**Fig. 3.**
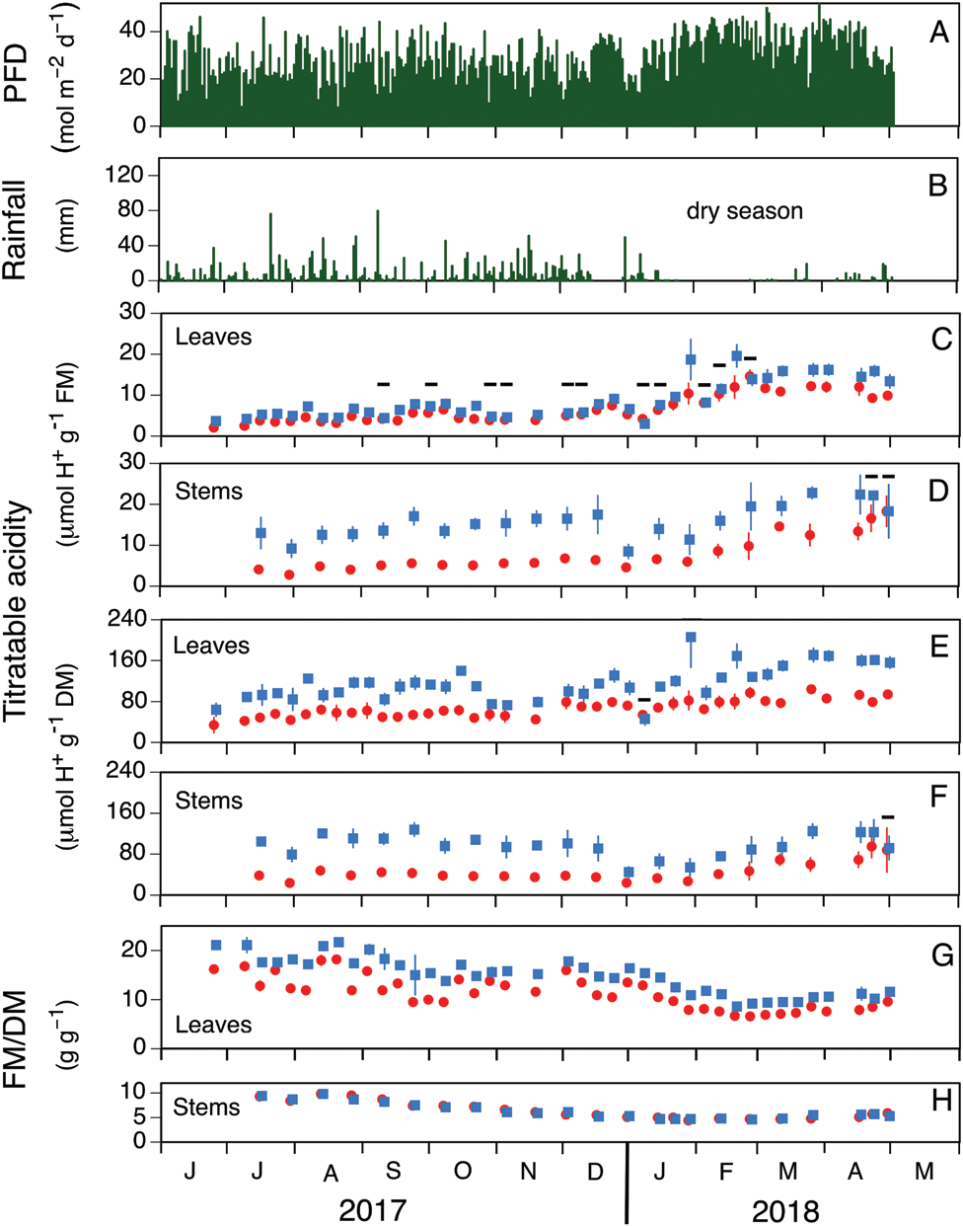
Seasonal changes in (A) photon flux density (PFD), (B) rainfall, (C–F) variation of titratable acidity at dusk (circles) and dawn (squares), and (G, H) variation in fresh:dry mass ratio (FM/DM) at dusk and dawn in leaves and stems of *Sesuvium portulacastrum* grown in soil supplemented with sea-salt. Plants were grown for most of the 2017 wet season, and as they transitioned into the 2018 dry season. Acidity levels are expressed per unit fresh mass for leaves (C) and stems (D) and per unit dry mass for leaves (E) and stems (F). Acidity and FM/DM values are means (±SD) (*n*=5; for leaves, each sample comprised eight leaves). In (C–F), horizontal bars indicate non-significant differences between dusk and dawn values (one-tailed *t*-test, *P*≤0.05).

The rainfall period lasted longer during the 2017/2018 season than during the 2016/2017 season of the first experiment (Fig. 3). Significant rainfall events extended into the middle of January 2018 (the wettest January since rainfall measurements started at the site 14 years previously). January 2018 rainfall was 80 mm, whereas January 2017 rainfall was 28 mm. In contrast to the previous experiment, plants survived the 2018 dry season.

As occurred in plants grown outdoors in non-saline soil, nocturnal acidification in *S. portulacastrum* was observed in both leaves and stems in plants grown outdoors under rainfall supplemented with 10% seawater (Fig. 3). During the rainy period, acidification in stems was consistently greater than in leaves, about 2-fold on a DM basis and 3-fold on a FM basis. Nevertheless, the absolute levels of nocturnal acidification in leaves and stems, irrespective on what basis they were expressed, were similar to those observed in plants grown during the previous year.

Again, during the wet season, leaves exhibited low levels of acidity on a FM basis that were significantly higher at dawn than at dusk on 22 of 28 wet-season days. On a DM basis, dawn values were higher on all wet-season days. Nocturnal acidification in leaves significantly increased during the dry season (collection dates from 27 January to 22 April 2018), from 1.4±0.8 μmol H^+^ g^−1^ FM (*n*=28 d) during the wet season to 3.8±3.0 μmol H^+^ g^−1^ FM (*n*=11 d) during the dry season (*P*≤0.05), and from 41±17 μmol H^+^ g^−1^ DM (*n*=28 d) during the wet season to 68±27 μmol H^+^ g^−1^ DM (*n*=11 d) during the dry season (*P*≤0.01). No significant dry-season increase in nocturnal acidification was observed in stems, either on a FM or a DM basis.

### Experiments with potted plants

**Fig. 4.**
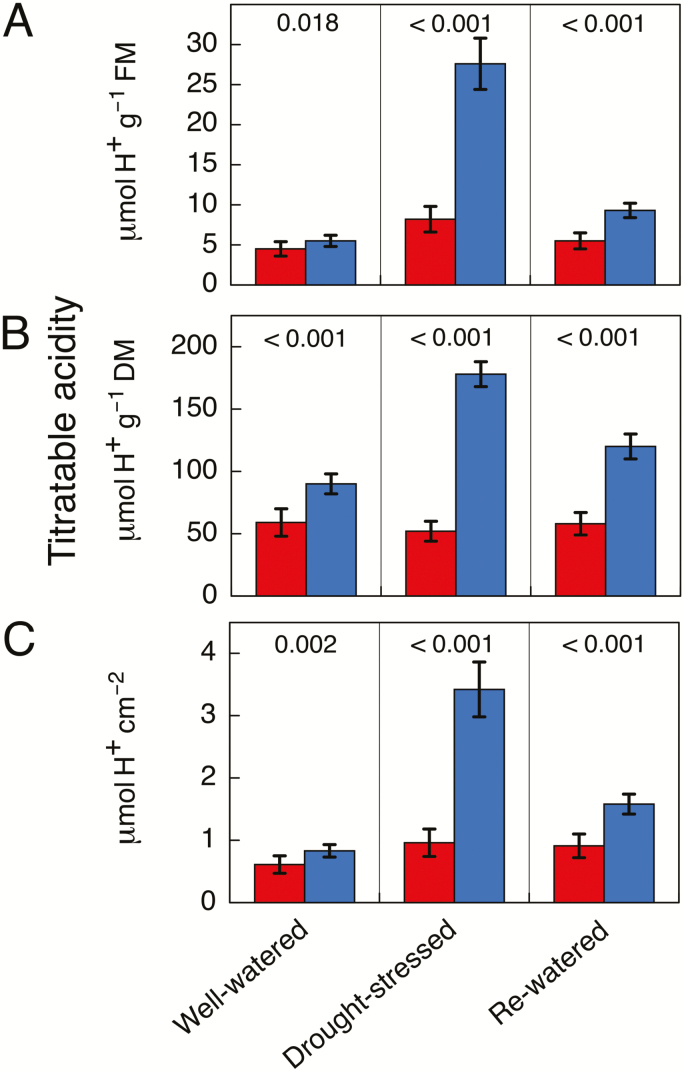
Titratable acidity at dusk (red) and dawn (blue) in leaves of *Sesuvium portulacastrum* grown in 3.5-l pots in soil supplemented with sea-salt underneath a rain shelter at 70% of natural sunlight. Samples were taken from eight plants. The experiment was performed from the end of October to early December 2017. Data are shown for well-watered plants, droughted plants (20 d without irrigation), and plants that had been droughted and rewatered (20 d with irrigation). The data are expressed on the basis of (A) fresh mass, (B) dry mass, and (C) leaf area, and are means (±SD) (*n*=8; each sample comprised eight leaves). *P*-values from one-tailed *t*-tests are shown.

Several pot experiments were conducted to study nocturnal acidification patterns in response to short-term drought stress. Leaves of well-irrigated plants supplied with either water or 10% seawater exhibited either no or a small nocturnal acidification. In the case of the experiment shown in Fig. 4, nocturnal acidification was below 2 μmol H^+^ g^−1^ FM under high initial soil moisture conditions. Following the imposition of water stress, the acid accumulation at night was stimulated ~19-fold. Upon rewatering, acidification was markedly reduced but was not eliminated, remaining at ~4 μmol H^+^ g^−1^ FM.

**Fig. 5.**
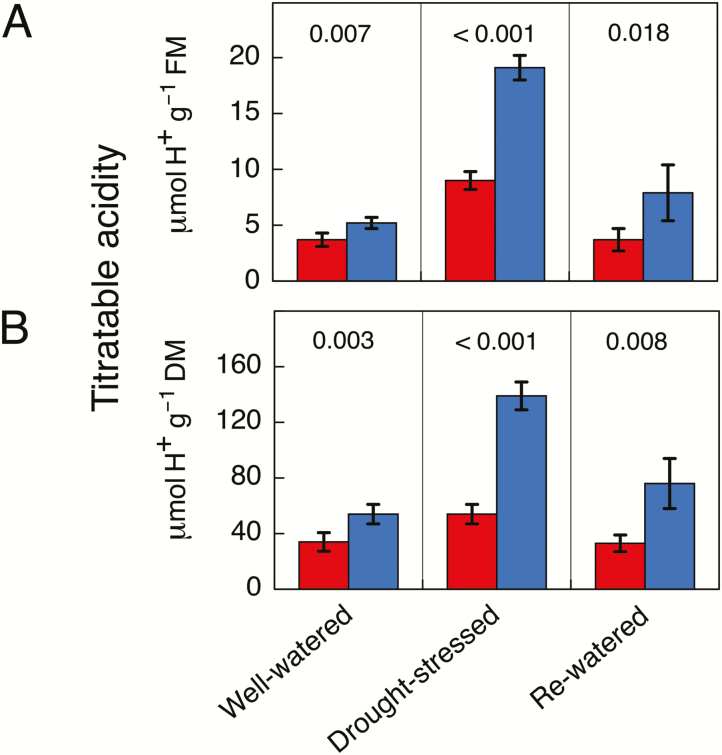
Titratable acidity at dusk (red) and dawn (blue) in stems of *Sesuvium portulacastrum* grown in 2.5-l pots in non-saline soil under full sunlight. The experiment was performed during January/February 2017. Samples were randomly taken from eight plants. Data are shown for well-watered plants, droughted plants (6 d without irrigation), and plants that had been droughted and rewatered (7 d with irrigation). The data are expressed on the basis of (A) fresh mass, and (B) dry mass, and are means (±SD) (*n*=4 stems; at a given time-point each stem was harvested from a different plant). *P*-values from one-tailed *t*-tests are shown.

In a separate experiment, stems of well-watered *S. portulacastrum* exhibited low levels of nocturnal acidification of less than 2 μmol H^+^ g^−1^ FM (Fig. 5). Following the cessation of watering, nocturnal acidification increased ~7-fold on a FM basis. When the plants were rewatered, the nocturnal accumulation was reduced markedly but was still present at a level that was ~3-fold greater than the values at the beginning of the experiment. The wet-dry-wet acidification patterns were similar when H^+^ was expressed on a DM or area basis for leaves or on a DM basis for stems. In all cases, a strong drought-induced up-regulation of nocturnal acid accumulation was observed, which was largely, albeit not fully, reversible.

**Fig. 6.**
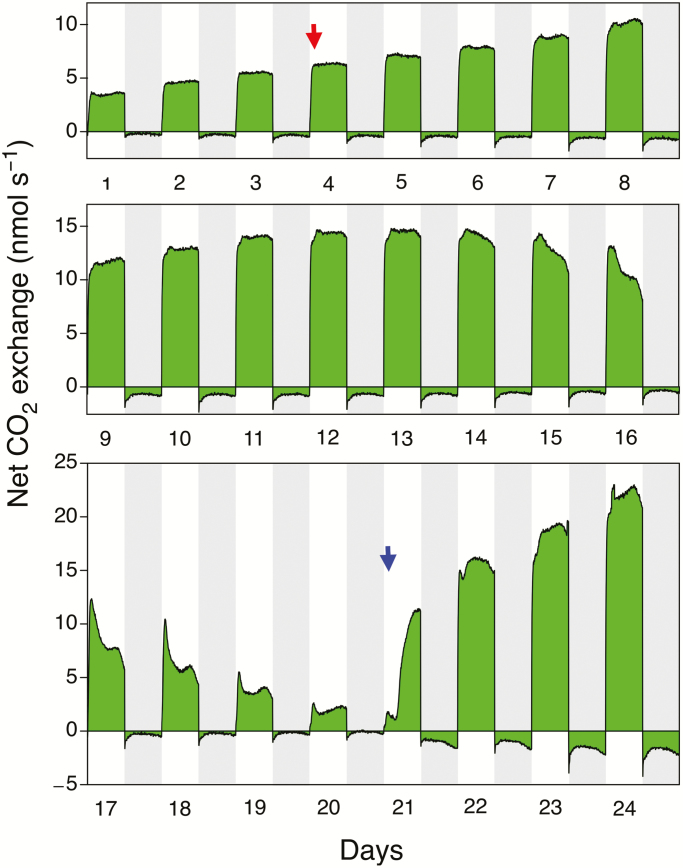
Net CO_2_ exchange over a 24-d period by the shoot of a *Sesuvium portulacastrum* plant growing in a 0.5-l pot containing non-saline soil. Watering was withheld on day 4 (red arrow) and recommenced on day 21 (blue arrow). The grey shaded areas represent the 12-h dark periods. Photon flux density was 700 µmol m^−2^ s^−1^ at the level of the shoot. On the last day of the experiment, the total leaf area was 6 cm^2^, the leaf dry mass was 0.129 g, and the stem dry mass was 0.024 g.

**Fig. 7.**
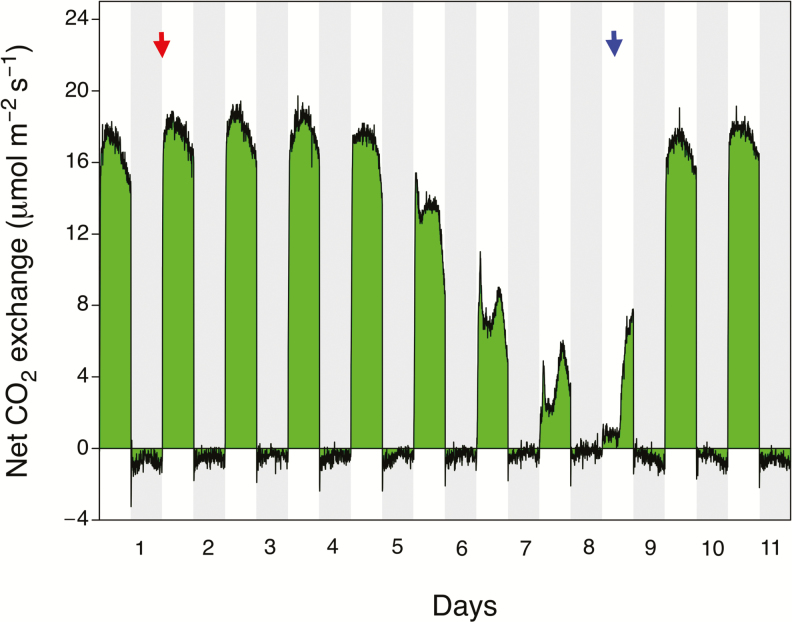
Net CO_2_ exchange over an 11-d period by an individual leaf of a *Sesuvium portulacastrum* plant growing in soil supplemented with sea-salt. Watering was withheld on day 2 (red arrow) and recommenced on day 9 (blue arrow). The grey shaded areas represent the 12-h dark periods. Photon flux density was 480 µmol m^−2^ s^−1^. Gas exchange was measured on a leaf area of 2.98 cm^2^.

Seven gas-exchange experiments were conducted with essentially identical results, two of which are depicted in Figs 6 and 7. For a well-watered plant of *S. portulacastrum*, net CO_2_ uptake by its shoot was restricted to the 12-h light period (Fig. 6). CO_2_ efflux was relatively constant throughout the night following an initial overshoot and period of equilibration to the night temperature, although a small curvature in the nocturnal CO_2_ release pattern was detectable with lower rates in the middle than at the end of the dark period. Following cessation of watering on day 4, CO_2_ uptake during the day continued to increase as the shoot continued to grow, utilizing water remaining in the pot. From day 14 onwards there was a progressive reduction in both CO_2_ uptake during the day and CO_2_ loss at night. Daytime CO_2_ exchange developed a prominent mid-day depression of uptake. Nocturnal CO_2_ exchange approached the CO_2_ compensation point but never transitioned to net CO_2_ gain.

Following rewatering, a recovery of CO_2_ uptake in the light was observed within 4 h and dark respiration increased during the subsequent night. Within 48 h after rewatering, the shoot exhibited a pattern of CO_2_ exchange similar to that observed in the original well-watered conditions. The kinetics of nocturnal CO_2_ efflux in the shoots of the rewatered plants were more curved than the efflux pattern in the original well-watered plants. The rate of CO_2_ loss was also greater. The daytime CO_2_ uptake rates following rewatering exceeded those at the onset of the experiment because the plants continued to grow inside the gas-exchange cuvette throughout the experiment, although at a reduced rate during the period of drought. The CO_2_ exchange pattern during a watering-droughting-rewatering cycle of a leaf of a plant, in this particular case irrigated with 10% seawater (Fig. 7), was similar to that of the shoot of a plant that was irrigated with water only (Fig. 6). The leaf of a well-watered plant exhibited net CO_2_ uptake during the day and net CO_2_ loss at night. At 3 d after watering ceased, daytime net CO_2_ exchange began to decrease, exhibiting a progressively larger mid-day depression of uptake. CO_2_ efflux at night also decreased, such that the CO_2_ compensation point was reached but net nocturnal CO_2_ uptake was not detected.

Within a few hours of rewatering on day 8, the rates of daytime CO_2_ uptake began to recover. During the following night, nocturnal CO_2_ loss gradually increased. By day 11, the patterns and extent of daytime CO_2_ uptake and night-time CO_2_ loss were similar to the patterns observed on day 1.

## Discussion

**Fig. 8.**
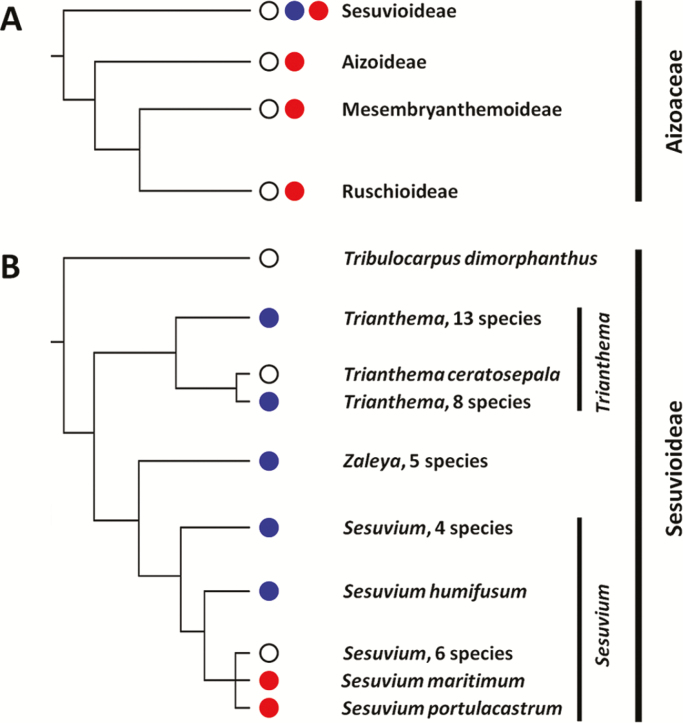
Distribution of CAM (red) and C_4_ (blue) within (A) the four sub-families of the Aizoaceae, and (B) genera of the sub-family Sesuvioides (adapted from Smith and Winter, 1996; Klak *et al.*, 2003; Muhaidat *et al.*, 2007; Bohley *et al.*, 2015, 2017, and the current study). Phylogenetic relationships in (A) are based upon analysis of three chloroplastic and nuclear loci (Klak *et al.*, 2003), and relationships in (B) are based upon analysis of five chloroplastic and nuclear loci (Bohley *et al.*, 2015). The open circles indicate plants with C_3_-type isotope values. Without measurements of acidification and gas exchange during a watering-droughting-rewatering regime, it cannot yet be determined whether such individuals exhibit C_3_*senso strictu*, low-level CAM, or are C_3_-C_4_ intermediates. In (B), the presence of CAM in *S. maritimum* is based upon nocturnal acidification reported during a single night for a plant growing in the field (Martin *et al.*, 1982).

The purpose of this study was to clarify whether CAM is present or absent in *S. portulacastrum*. To this end, plants grown outdoors in the ground or potted plants grown under more controlled conditions were subjected to a range of conditions, including supplementation with NaCl and drought stress. Under most conditions, small nocturnal increases in acid content were measured in both leaves and stems, suggesting that CAM is present in *Sesuvium* and thus in the Sesuvioideae, the only sub-family of the Aizoaceae for which CAM had not yet been conclusively documented (Fig. 8). Given the ability to perform low-level CAM, *S. portulacastrum* joins a growing number of species that cannot be considered C_3_ plants *sensu strictu*, although they obtain CO_2_ principally via the C_3_-pathway.

Whilst CAM photosynthesis is present in *S. portulacastrum*, in comparison to C_3_ photosynthesis, the contribution of nocturnal CO_2_ assimilation to the carbon gain of the plant is extremely small. Whole-leaf carbon isotope values of plants in the field of generally around –24 to –26‰ (Winter *et al.*, 1981; Lüttge *et al.*, 1989; Medina *et al.*, 2008; Bohley *et al.*, 2015), indicate that, averaged over the life of the plant, the vast majority of carbon is assimilated by C_3_ photosynthesis during the light (Winter and Holtum, 2002). In the gas-exchange time-courses for shoots and leaves shown in Figs 6 and 7 (as well as in the additional experiments for which data are not shown), the rates of dark CO_2_ fixation were not high enough to compensate fully for respiratory CO_2_ loss. CO_2_ exchange at night approached but did not exceed the CO_2_ compensation point.

Similar levels of nocturnal acidification accumulated in plants grown in outdoor raised garden plots and in plants grown in pots under more controlled conditions. Acidification was detected effectively throughout the life of the plants, even during the wet season, although acidification levels in leaves were greater during the dry season. In contrast to the evidence from the outdoor experiments, examination of the pot experiment acidification data (Figs 4, 5) would suggest that most, if not all, of the CAM expression in leaves was facultative as acidification in non-stressed leaves was extremely low. The evidence for mostly facultative CAM in the pot experiments with controlled irrigation and for longer-term low-level CAM in the outdoor experiments is not necessarily contradictory. Observation of leaves of *S. portulacastrum* on outwardly healthy, fast-growing plants growing in the outdoor plots subject to natural daylight during the wet season indicated that the relatively elastic leaves (low elastic modulus) often appeared flaccid in the afternoons. The resultant leaf water deficit was evidently sufficient to lead to low-level CAM that persisted throughout the experiment. A similar phenomenon has been reported to induce CAM in leaves of well-watered plants of the facultative CAM plant *M. crystallinum* (Winter and Holtum, 2007), especially in plants grown under non-saline conditions.

Observations in the two outdoor experiments of increased nocturnal acidification in leaves during the dry season are consistent with a strong facultative CAM component in leaves, while the absence of a consistent dry-season increase in nocturnal stem acidification suggests that low-level CAM in stems has a substantial constitutive component. The ability of *S. portulacastrum* to maintain nocturnal acidification levels well into the dry season, even as FM/DM ratios fell below 5 for stems and below 10 in succulent leaves, provides an indication of the relative drought-tolerance of the species. Indeed, plants growing on a saline plain in northern Venezuela have been reported to operate at xylem tensions of as low as –5.6 MPa (Lüttge *et al.*, 1989).

CAM is now documented in all four sub-families of the Aizoaceae (Fig. 8A). Pronounced CAM, undoubtedly overwhelmingly constitutive, is present in the sub-families Azooideae, Mesembryanthemoideae, and Ruschioideae (Smith and Winter, 1996), and facultative CAM is present in the Mesembryanthemoideae [e.g. *Mesembranthemum crystallinum* (Winter and Holtum, 2007) and *Aptenia cordifolia* (Treichel, 1975)] and now in the Sesuvioideae (*S. portulacastrum*). All of the sub-types of CAM, constitutive, facultative, pronounced and weak, are also known in the only other clade of the Caryophyllales with CAM plants, the sub-order Portulacineae. In the portullugo (Portulacineae plus Molluginaceae; Ogburn and Edwards, 2010; Edwards and Ogburn, 2012), pronounced CAM predominates in most genera within the Cactaceae and Didiereaceae (*Alluaudia*, *Alluaudiopsis*, *Decarya*, *Didierea*), whereas weak and facultative CAM are more commonly expressed in the Anacampserotaceae (Guralnick and Jackson, 2001; Winter and Holtum, 2017), *Portulacaria* within the Didiereaceae (Ting and Hanscom 1977; Guralnick and Ting, 1986), Montiaceae (Winter and Holtum, 2011; Martin *et al.*, 1988), Talinaceae (Herrera *et al.*, 1991), Basellaceae (Holtum *et al.*, 2018), and in the C_4_ and C_3_-C_4_ Portulacaceae (Koch and Kennedy, 1980; Winter and Holtum, 2017; K. Winter *et al.*, unpublished results). In the thin-leaved Molluginaceae, C_4_ and C_3_ are present but CAM has not been reported (Christin *et al.*, 2011).

CAM lineages and C_4_ lineages tend to cluster in certain regions of the angiosperm phylogenetic tree (Sage *et al.*, 2011*a*; Edwards and Ogburn, 2012; Christin *et al.*, 2015), with particularly closely related CAM and C_4_ lineages identified within the Aizoaceae, Euphorbiaceae, and Portulacineae (Klak *et al.*, 2003, 2017; Sage *et al.*, 2011*b*; Horn *et al.*, 2012, 2014; Ocampo *et al.*, 2013; Christin *et al.*, 2014). Following the discovery of CAM and C_4_ photosynthesis in *Euphorbia* and *Portulaca*, *Sesuvium* becomes only the third genus known to contain both types of carbon-concentrating photosynthesis.

In the Sesuvioides, the most basal of the sub-families in the Aizoaceae, CAM is reported here in *Sesuvium*, the most derived clade (Fig. 8B). C_4_ is present in *Zaleya* and *Trianthema* (Muhaidat *et al.*, 2007; Koteyeva *et al.*, 2015), whereas neither C_4_ nor CAM have been reported in the most basal clade, *Tribulocarpus* (Bohley *et al.*, 2015, 2017). Succulence is present in *Sesuvium*, *Trianthema*, and to a lesser extent in *Zaleya* and *Tribulocarpus*.

CAM and C_4_ are present in *Sesuvium* but they are yet to be demonstrated in the same species. The genus circumscribes a C_3_ clade and a C_4_ clade (Bohley *et al*. 2015, 2017). Both *S. portulacastrum* and the closely related *S. maritimum* studied by Martin *et al.* (1982) are in the C_3_ clade. It would be informative to explore whether CAM is present within members of C_4_*Sesuvium* clade. In contrast to what is currently known about *Sesuvium*, in *Portulaca* CAM is expressed within at least three clades of C_4_ species and thus, in those species with CAM, it is co-expressed with C_4_ in the same plant (Koch and Kennedy, 1980; Holtum *et al.*, 2017*b*; Winter and Holtum, 2017). In *P. cryptopetala*, facultative CAM is present in a C_3_-C_4_ intermediate species that exhibits a C_2_-cycle (Muhaidat *et al.*, 2007; Vozsenskaya *et al.*, 2010, 2017; K. Winter *et al.*, unpublished results). Within the genus *Euphorbia*, which contains C_3_, C_4_, and CAM species (Webster *et al.*, 1975; Sage *et al.*, 2011*b*; Yang and Berry, 2011), there are no reports as yet of CAM within the C_4_ clade (subg. *Chamaesyce*), although it appears to have repeatedly evolved alongside succulence from C_3_ lineages (Horn *et al.*, 2014). Together, the *Euphorbia*, *Portulaca*, and *Sesuvium* lineages with their differing evolutionary histories of CAM and C_4_ photosynthesis provide promise for exploring the biochemical, anatomical, and ecological trajectories that favour the selection of one carbon-concentrating mechanism over the other, or permit the co-existence of both.

The ecological significance of the low-level contribution of carbon contributed by CAM to *S. portulacastrum* is as yet unassessed, but as is evident from the outdoor experiments described here, low-level and facultative CAM can be expressed over extensive periods. As a species that is a coloniser of the fringes of saline flats, *S. portulacastrum* probably spends much of its life growing slowly under conditions of salinity, drought stress, high light, and high temperatures, as evidenced by the vividly red-coloured fleshy stems and wilted leaves frequently exhibited by plants in the field. Under such conditions one might expect CO_2_ assimilation in the light to be constrained by water limitation. The reductions in water loss and respiratory carbon loss enabled by the low-level CAM, an ability to accumulate compatible solutes, and to accumulate the anion oxalate (Lüttge *et al.* 1989), coupled with other physiological mechanisms (Lokhande *et al.*, 2013*b*), presumably assist plants to cope with the stresses imposed by the environment it inhabits.

In terms of carbon-isotope composition and gas-exchange pattern, *S. portulacastrum* is C_3_-like. Yet our multiple measurements of plants in pots and long-term measurements in the field demonstrate that *S. portulacastrum* consistently exhibits CAM in both leaves and stems. Since the levels of nocturnal acidification are low and easy to overlook, caution is required when assessing such plants for photosynthetic pathway. This study also highlights the care required when selecting C_3_ control species in evolutionary studies of lineages in which CAM or C_4_ has evolved. CAM and C_4_ origins may not always represent completely independent evolutionary phenomena, and may partially share evolutionary trajectories in that one photosynthetic type can be co-opted to evolve the other (Christin *et al.*, 2014).

## References

[CIT0001] Alonso-Cantabrana H, von Caemmerer S 2016 Carbon isotope discrimination as a diagnostic tool for C_4_ photosynthesis in C_3_–C_4_ intermediate species. Journal of Experimental Botany 67, 3109–3121. 26862154 10.1093/jxb/erv555 PMC4867892

[CIT0002] Arakaki M, Christin P-A, Nyffeler R, Lendel A, Eggli U, Ogburn RM, Spriggs E, Moore MJ, Edwards EJ 2011 Contemporaneous and recent radiations of the world’s major succulent plant lineages. Proceedings of the National Academy of Sciences, USA 108, 8379–8384. 10.1073/pnas.1100628108 PMC3100969 21536881

[CIT0003] Bittrich V 1990 Systematic studies in Aizoaceae. Mitteilungen aus dem Institut für Allgemeine Botanik in Hamburg23b, 491–507.

[CIT0004] Bohley K, Joos O, Hartmann H, Sage R, Liede-Schumann S, Kadereit G 2015 Phylogeny of Sesuvioideae (Aizoaceae) – Biogeography, leaf anatomy and the evolution of C_4_ photosynthesis. Perspectives in Plant Ecology, Evolution and Systematics 17, 116–130.

[CIT0005] Bohley K, Winter PJD, Kadereit G 2017 A revision of *Sesuvium* (Aizoaceae, Sesuvioideae). Systematic Botany 42, 124–147.

[CIT0006] Carolin RC, Jacobs SWL, Vesk M 1978 Kranz cells and mesophyll in the Chenopodiales. Australian Journal of Botany 26, 683–698.

[CIT0007] Christin PA, Arakaki M, Osborne CP, et al 2014 Shared origins of a key enzyme during the evolution of C_4_ and CAM metabolism. Journal of Experimental Botany 65, 3609–3621. 24638902 10.1093/jxb/eru087 PMC4085957

[CIT0008] Christin PA, Arakaki M, Osborne CP, Edwards EJ 2015 Genetic enablers underlying the clustered evolutionary origins of C_4_ photosynthesis in angiosperms. Molecular Biology and Evolution 32, 846–858. 25582594 10.1093/molbev/msu410

[CIT0009] Christin PA, Sage TL, Edwards EJ, Ogburn RM, Khoshravesh R, Sage RF 2011 Complex evolutionary transitions and the significance of C_3_-C_4_ intermediate forms of photosynthesis in Molluginaceae. Evolution 65, 643–660. 20955197 10.1111/j.1558-5646.2010.01168.x

[CIT0010] Edwards EJ, Ogburn M 2012 Angiosperm responses to a low-CO_2_ world: CAM and C_4_ photosynthesis as parallel evolutionary trajectories. International Journal of Plant Sciences 173, 724–733.

[CIT0011] Gilbert MG, Hartmann HEK, Edwards S 2000 Aizoaceae. In: Edwards S, Tadesse S, Demissew S, Hedberg I, eds. Flora of Ethiopia & Eritrea. Vol. 2 Addis Ababa and Uppsala: National Herbarium/Uppsala University, 240–248.

[CIT0012] Gonçalves ML 1978 Aizoaceae. In Launert E, ed. Flora Zambesiaca. Vol. 4 London: Flora Zambesiaca Managing Committee, 508–521.

[CIT0013] Guralnick LJ, Jackson MD 2001 The occurrence and phylogenetics of crassulacean acid metabolism in the Portulacaceae. International Journal of Plant Sciences 162, 257–262.

[CIT0014] Guralnick LJ, Ting IP 1986 Seasonal response to drought and rewatering in *Portulacaria afra* (L.) Jacq. Oecologia 70, 85–91. 28311290 10.1007/BF00377114

[CIT0015] Hartmann HEK 2017 Illustrated handbook of succulent plants: Aizoaceae. 2nd edn. Heidelberg: Springer.

[CIT0016] Hernández-Ledesma P, Berendsohn WG, Borsch T, et al 2015 A taxonomic backbone for the global synthesis of species diversity in the angiosperm order Caryophyllales. Willdenowia 45, 281–383.

[CIT0017] Herrera A, Delgado J, Paraguatey I 1991 Occurrence of inducible crassulacean acid metabolism in leaves of *Talinum triangulare* (Portulacaceae). Journal of Experimental Botany 42, 493–499.

[CIT0018] Heyduk K, Burrell N, Lalani F, Leebens-Mack J 2016 Gas exchange and leaf anatomy of a C_3_-CAM hybrid, *Yucca gloriosa* (Asparagaceae). Journal of Experimental Botany 67, 1369–1379. 26717954 10.1093/jxb/erv536 PMC4762382

[CIT0019] Holtum JAM, Hancock LP, Edwards EJ, Winter K 2017a Facultative CAM photosynthesis (crassulacean acid metabolism) in four species of *Calandrinia*, ephemeral succulents of arid Australia. Photosynthesis Research 134, 17–25. 28871459 10.1007/s11120-017-0359-x

[CIT0020] Holtum JAM, Hancock LP, Edwards EJ, Winter K 2017b Optional use of CAM photosynthesis in two C_4_ species, *Portulaca cyclophylla* and *Portulaca digyna*. Journal of Plant Physiology 214, 91–96. 28511087 10.1016/j.jplph.2017.01.010

[CIT0021] Holtum JAM, Hancock LP, Edwards EJ, Winter K 2018 Crassulacean acid metabolism (CAM) in the Basellaceae (Caryophyllales). Plant Biology 20, 409–414. 29369469 10.1111/plb.12698

[CIT0022] Holtum JAM, Winter K 1999 Degrees of crassulacean acid metabolism in tropical epiphytic ferns. Australian Journal of Plant Physiology 26, 749–757.

[CIT0023] Holtum JA, Winter K 2003 Photosynthetic CO_2_ uptake in seedlings of two tropical tree species exposed to oscillating elevated concentrations of CO_2_. Planta 218, 152–158. 12905026 10.1007/s00425-003-1089-1

[CIT0024] Horn JW, van Ee BW, Morawetz JJ, Riina R, Steinmann VW, Berry PE, Wurdack KJ 2012 Phylogenetics and the evolution of major structural characters in the giant genus *Euphorbia* L. (Euphorbiaceae). Molecular Phylogenetics and Evolution 63, 305–326. 22273597 10.1016/j.ympev.2011.12.022

[CIT0025] Horn JW, Xi Z, Riina R, Peirson JA, Yang Y, Dorsey BL, Berry PE, Davis CC, Wurdack KJ 2014 Evolutionary bursts in *Euphorbia* (Euphorbiaceae) are linked with photosynthetic pathway. Evolution 68, 3485–3504. 25302554 10.1111/evo.12534

[CIT0026] Klak C, Hanacek P, Bruyns PV 2017 Disentangling the Aizooideae: new generic concepts and a new subfamily in Aizoaceae. Taxon 66, 1147–1170.

[CIT0027] Klak C, Khunou A, Reeves G, Hedderson T 2003 A phylogenetic hypothesis for the Aizoaceae (Caryophyllales) based on four plastid DNA regions. American Journal of Botany 90, 1433–1445. 21659095 10.3732/ajb.90.10.1433

[CIT0028] Koch K, Kennedy RA 1980 Characteristics of crassulacean acid metabolism in the succulent C_4_ dicot, *Portulaca oleracea* L. Plant Physiology 65, 193–197. 16661159 10.1104/pp.65.2.193 PMC440296

[CIT0029] Koteyeva NK, Voznesenskaya EV, Edwards GE 2015 An assessment of the capacity for phosphoenolpyruvate carboxykinase to contribute to C_4_ photosynthesis. Plant Science 235, 70–80. 25900567 10.1016/j.plantsci.2015.03.004

[CIT0030] Lokhande VH, Gor BK, Desai NS, Nikam TD, Suprasanna P 2013a Biochemical and physiological adaptations of the halophyte *Sesuvium portulacastrum* (L.) L., (Aizoaceae) to salinity. Archives of Agronomy and Soil Science 59, 1373–1391.

[CIT0031] Lokhande VH, Gor BK, Desai NS, Nikam TD, Suprasanna P 2013b *Sesuvium portulacastrum*, a plant for drought, salt stress, sand fixation, food and phytoremediation. A review. Agronomy for Sustainable Development 33, 329–348.

[CIT0032] Lonard RI, Judd FW 1997 The biological flora of coastal dunes and wetlands. *Sesuvium portulacastrum* (L.) L. Journal of Coastal Research 13, 96–104.

[CIT0033] Lüttge U, Popp M, Medina E, Cram WJ, Diaz M, Griffiths H, Lee HSJ, Schäfer C, Smith JAC, Stimmel KH 1989 Ecophysiology of xerophytic and halophytic vegetation of a coastal alluvial plain in northern Venezuela. V. The *Batis maritima*-*Sesuvium portulacastrum* vegetation unit. New Phytologist 111, 283–291. 10.1111/j.1469-8137.1989.tb00692.x 33874251

[CIT0034] Martin CE, Higley M, Wang WZ 1988 Ecophysiological significance of CO_2_-recycling via Crassulacean acid metabolism in *Talinum calycinum* Engelm. (Portulacaceae). Plant Physiology 86, 562–568. 16665946 10.1104/pp.86.2.562 PMC1054523

[CIT0035] Martin CE, Lubbers AE, Teeri JA 1982 Variability in crassulacean acid metabolism: a survey of North Carolina succulent species. Botanical Gazette 143, 491–497.

[CIT0036] Medina E, Francisco AM, Wingfield R, Casañas OL 2008 Halofitismo en plantas de la costa caribe de Venezuela: halófitas y halotolerantes. Acta Botanica Venezuelica 31, 49–80.

[CIT0037] Muhaidat R, Sage RF, Dengler NG 2007 Diversity of Kranz anatomy and biochemistry in C_4_ eudicots. American Journal of Botany 94, 362–381. 21636407 10.3732/ajb.94.3.362

[CIT0038] Ocampo G, Koteyeva NK, Voznesenskaya EV, Edwards GE, Sage TL, Sage RF, Columbus JT 2013 Evolution of leaf anatomy and photosynthetic pathways in Portulacaceae. American Journal of Botany 100, 2388–2402. 24259525 10.3732/ajb.1300094

[CIT0039] Ogburn RM, Edwards EJ 2010 The ecological water-use strategies of succulent plants. Advances in Botanical Research 55, 179–225.

[CIT0040] Osmond CB 1978 Crassulacean acid metabolism: a curiosity in context. Annual Review of Plant Physiology 29, 379–414.

[CIT0041] Reef R, Slot M, Motro U, Motro M, Motro Y, Adame MF, Garcia M, Aranda J, Lovelock CE, Winter K 2016 The effects of CO_2_ and nutrient fertilisation on the growth and temperature response of the mangrove *Avicennia germinans*. Photosynthesis Research 129, 159–170. 27259536 10.1007/s11120-016-0278-2

[CIT0042] Sage RF, Christin P-A, Edwards EJ 2011a The C_4_ plant lineages of planet Earth. Journal of Experimental Botany 62, 3155–3169. 21414957 10.1093/jxb/err048

[CIT0043] Sage TL, Sage RF, Vogan PJ, Rahman B, Johnson DC, Oakley JC, Heckel MA 2011b The occurrence of C_2_ photosynthesis in *Euphorbia* subgenus *Chamaesyce* (Euphorbiaceae). Journal of Experimental Botany 62, 3183–3195. 21459765 10.1093/jxb/err059

[CIT0044] Smith JAC, Winter K 1996 Taxonomic distribution of crassulacean acid metabolism. In: Winter K, Smith JAC, eds. Crassulacean acid metabolism. Berlin, Heidelberg: Springer, 427–436.

[CIT0045] Thulin M, Thiede J, Liede-Schumann S 2012 Phylogeny and taxonomy of *Tribulocarpus* (Aizoaceae): a paraphyletic species and an adaptive shift from zoochorous trample burrs to anemochorous nuts. Taxon 61, 55–66.

[CIT0046] Ting IP 1989 Photosynthesis of arid and subtropical succulent plants. Aliso 12, 387–406.

[CIT0047] Ting IP, Hanscom Z 1977 Induction of acid metabolism in *Portulacaria afra*. Plant Physiology 59, 511–514. 16659882 10.1104/pp.59.3.511 PMC542433

[CIT0048] Treichel S 1975 Crasulaceensäuerstoffwechsel bei einem salztoleranten Vertreter der Aizoaceae: *Aptenia cordifolia*. Plant Science Letters 4, 141–144.

[CIT0049] von Willert DJ, Eller BM, Werger MJA, Brinckmann E, Ihlenfeldt HD 1992 Life strategies of succulents in deserts. Cambridge: Cambridge University Press.

[CIT0050] von Willert DJ, Thomas DA, Lobin W, Curdts E 1977 Ecophysiological investigations in the family of the Mesembryanthemaceae. Oecologia 29, 67–76. 28308807 10.1007/BF00345363

[CIT0051] Voznesenskaya EV, Koteyeva NK, Edwards GE, Ocampo G 2010 Revealing diversity in structural and biochemical forms of C_4_ photosynthesis and a C_3_-C_4_ intermediate in genus *Portulaca* L. (Portulacaceae). Journal of Experimental Botany 61, 3647–3662. 20591900 10.1093/jxb/erq178 PMC2921202

[CIT0052] Voznesenskaya EV, Koteyeva NK, Edwards GE, Ocampo G 2017 Unique photosynthetic phenotypes in *Portulaca* (Portulacaceae): C_3_-C_4_ intermediates and NAD-ME C_4_ species with Pilosoid-type Kranz anatomy. Journal of Experimental Botany 68, 225–239. 27986845 10.1093/jxb/erw393 PMC5853368

[CIT0053] Webster GL, Brown WV, Smith BN 1975 Systematics of photosynthetic carbon fixation pathways in Euphorbia. Taxon 24, 27–33.

[CIT0054] Winter K, Garcia M, Holtum JA 2008 On the nature of facultative and constitutive CAM: environmental and developmental control of CAM expression during early growth of *Clusia*, *Kalanchoë*, and *Opuntia*. Journal of Experimental Botany 59, 1829–1840. 18440928 10.1093/jxb/ern080

[CIT0055] Winter K, Holtum JA 2002 How closely do the δ^13^C values of crassulacean acid metabolism plants reflect the proportion of CO_2_ fixed during day and night? Plant Physiology 129, 1843–1851. 12177497 10.1104/pp.002915 PMC166772

[CIT0056] Winter K, Holtum JA 2007 Environment or development? Lifetime net CO_2_ exchange and control of the expression of crassulacean acid metabolism in *Mesembryanthemum crystallinum*. Plant Physiology 143, 98–107. 17056756 10.1104/pp.106.088922 PMC1761986

[CIT0057] Winter K, Holtum JAM 2011 Induction and reversal of crassulacean acid metabolism in *Calandrinia polyandra*: effects of soil moisture and nutrients. Functional Plant Biology 38, 576–582. 10.1071/FP11028 32480910

[CIT0058] Winter K, Holtum JAM 2014 Facultative CAM plants: powerful tools for unravelling the functional elements of CAM photosynthesis. Journal of Experimental Botany 65, 3425–3441. 24642847 10.1093/jxb/eru063

[CIT0059] Winter K, Holtum JAM 2015 Cryptic crassulacean acid metabolism (CAM) in *Jatropha curcas* L. Functional Plant Biology 42, 711–717. 10.1071/FP15021 32480714

[CIT0060] Winter K, Holtum JAM 2017 CO_2_-exchange patterns demonstrate facultative CAM photosynthesis (crassulacean acid metabolism) in four small C_3_ and C_4_ leaf-succulents. Australian Journal of Botany 65, 103–108.

[CIT0061] Winter K, Holtum JA, Smith JA 2015 Crassulacean acid metabolism: a continuous or discrete trait? New Phytologist 208, 73–78. 25975197 10.1111/nph.13446

[CIT0062] Winter K, Osmond CB, Pate JS 1981 Coping with salinity. In: Pate JS, McComb AJ, eds. The biology of Australian plants. Perth: University of Western Australia Press, 88–113.

[CIT0063] Yang Y, Berry PE 2011 Phylogenetics of the Chamaesyce clade (*Euphorbia*, Euphorbiaceae): reticulate evolution and long-distance dispersal in a prominent C_4_ lineage. American Journal of Botany 98, 1486–1503. 21875975 10.3732/ajb.1000496

